# Construction of an abnormal glycosylation risk model and its application in predicting the prognosis of patients with head and neck cancer

**DOI:** 10.1038/s41598-023-50092-6

**Published:** 2024-01-15

**Authors:** Yihan Gao, Wenjing Li, Haobing Guo, Yacui Hao, Lili Lu, Jichen Li, Songlin Piao

**Affiliations:** 1 Department of Oral and Maxillofacial Surgery, The First Affiliated Hospital of Harbin Medical University, Harbin, 150000 China; 2 School of Stomatology, Harbin Medical University, Harbin, 150000 China; 3 College of Animal Science, Zhejiang University, Hangzhou, 310058 China

**Keywords:** Cancer, Head and neck cancer, Oral cancer

## Abstract

Head and neck squamous cell carcinoma (HNSCC) is the most common malignant tumor of the head and neck, and the incidence rate is increasing year by year. Protein post-translational modification, recognized as a pivotal and extensive form of protein modification, has been established to possess a profound association with tumor occurrence and progression. This study employed bioinformatics analysis utilizing transcriptome sequencing data, patient survival data, and clinical data from HNSCC to establish predictive markers of genes associated with glycosylation as prognostic risk markers. The R procedure WGCNA was employed to construct a gene co-expression network using the gene expression profile and clinical characteristics of HNSCC samples. Multiple Cox Proportional Hazards Regression Model (Cox regression) and LASSO analysis were conducted to identify the key genes exhibiting the strongest association with prognosis. A risk score, known as the glycosylation-related genes risk score (GLRS), was subsequently formulated utilizing the aforementioned core genes. This scoring system facilitated the classification of samples into high-risk and low-risk categories, thereby enabling the prediction of patient prognosis. The association between GLRS and clinical variables was examined through both univariate and multivariate Cox regression analysis. The validation of six core genes was accomplished using quantitative real-time polymerase chain reaction (qRT-PCR). The findings demonstrated noteworthy variations in risk scores among subgroups, thereby affirming the efficacy of GLRS in prognosticating patient outcomes. Furthermore, a correlation has been observed between the risk-scoring model and immune infiltration. Moreover, significant disparities exist in the expression levels of diverse immune checkpoints, epithelial-mesenchymal transition genes, and angiogenic factors between the high and low-risk groups.

## Introduction

Protein post-translational modification (PTM) plays a crucial role in numerous biological processes by relying on glycoproteins and glycosylation, which contribute to essential physiological activities such as augmenting protein stability, impeding protein degradation, enhancing protein solubility, and regulating protein functionality. Post-translational modification (PTM) is a significant and recurrent alteration that occurs in proteins subsequent to translation, with glycosylation being a prevalent form of PTM in over half of eukaryotic proteins. This process plays a crucial role in various cellular functions such as intercellular recognition, regulation, signaling, immune response, cellular transformation, and disease progression. It is noteworthy that protein glycosylation also holds paramount importance in tumor metastasis, oncogenic effects, tumor microenvironment, tumor growth, and proliferation, as well as angiogenesis^1^.

Head and neck tumors rank as the sixth most prevalent form of malignancy worldwide. Factors such as advancing age, tumor stage, smoking, and alcohol consumption are associated with reduced overall survival rates for individuals with head and neck tumors^2^. Furthermore, head and neck tumors exhibit the highest number of primary sites, pathological types, and complex anatomical connections among all tumors in the human body. Head and neck squamous carcinoma (HNSCC) accounts for over 95% of all head and neck cancers^3^. The advent of targeted pharmaceuticals has emerged as a novel therapeutic strategy for addressing HNSCC, while targeted immunotherapy has introduced innovative perspectives for clinical intervention^4^, However, given the intricate association between HNSCC progression and genetic and protein factors, as well as the interdependence between molecular markers and treatment response, it becomes imperative to investigate efficacious biomarkers that facilitate the judicious administration of tailored drugs to diverse patients. Consequently, the development of personalized treatment regimens becomes an indispensable pursuit. Consequently, the present study aims to investigate efficacious biomarkers for targeted drug utilization in diverse patient cohorts and to devise personalized therapeutic strategies.

The proliferation of bioinformatics has significantly bolstered the utilization of gene expression profiling for the identification of novel biomarkers. Primarily, bioinformatics expedites researchers' acquisition of extensive datasets, enabling them to access diverse research sources and employ cost-free databases and tools to scrutinize the data^5^. Current biological research is focused on studying the distinct functions and attributes of individual genes, transcripts, and proteins. While this approach is instrumental in elucidating the molecular mechanisms underlying life, it inherently offers limited insights into biological systems and restricts a thorough and comprehensive understanding of their overall behavior. Biological networks serve as a valuable tool for visualizing the interconnectedness of functional components within biological systems, thereby facilitating the comprehensive study of organism characteristics at a systemic level^6^.

Weighted gene co-expression network analysis (WGCNA) technique proves instrumental in investigating gene expression variations across multiple samples. By enabling the clustering and formation of gene modules based on shared expression patterns, WGCNA allows for the analysis of associations between these modules and specific traits of interest^7^. In this study, it was postulated that the gene expression network adhered to a scale-free distribution, and a network of co-expressed genes was constructed using the Weighted Gene Co-expression Network Analysis (WGCNA) algorithm. Subsequently, the coefficient of variation was computed for each node to construct agglomerative trees. Additionally, genes exhibiting high similarity were grouped into the same modules, while those displaying low resemblance were assigned to separate modules, and these modules were visually represented. This study employed WGCNA analysis on glycosylated genes obtained from the TCGA and HNSC cohorts. Subsequently, glycosylated genes within significant modules were chosen to develop a risk score model comprising 16 prognostic markers for predicting patient prognosis. The model's effectiveness remained consistent when assessing and validating the training and validation sets. Additionally, the model groups exhibited associations with various clinical characteristics, immune infiltration, and the expression of multiple immune checkpoint genes. There were notable disparities observed in the expression of EMT genes and angiogenic factors between the high- and low-risk groups. This investigation has provided innovative insights and objectives for future studies and therapeutic interventions in the context of HNSCC, by identifying prognostic markers that can predict the prognosis of patients with this condition. Supplementary Fig. 1 show the flowchart of this study.

## Materials and methods

### Data download and pre-processing

The FPKM expression profile data and survival information of Head and Neck Squamous Cell Carcinoma (HNSCC) from The Cancer Genome Atlas (TCGA) were obtained using the R package TCGA bio links (https://portal.gdc.cancer.gov/) for the purpose of constructing the model. Open the Genecards website and search for Glycosylation (https://www.genecards.org/Search/Keyword?queryString=Glycosylation). Then export to Excel and select genes greater than the median score based on the score, we screened 3785 glycosylation-related genes from the Genecards database. Subsequently, a weighted gene co-expression network analysis was conducted, resulting in the identification of 749 genes. Furthermore, a single-factor Cox regression analysis was performed, leading to the identification of 38 genes associated with overall survival. Ultimately, a LASSO linear regression method was employed to screen and select 16 prognostic marker genes, which were utilized to construct a prognostic model. Furthermore, the clinical and expression data for GSE65858 and GSE41613 were obtained from the Gene Expression Omnibus (GEO) database (https://www.ncbi.nlm.nih.gov/geo/) to serve as the validation set for the risk model^8^.

The dbEMT database represents a pioneering effort in compiling a comprehensive collection of EMT-related genes^9^, thereby providing a valuable reference tool for investigating the underlying biological processes associated with EMT, particularly in the context of cancer development and metastasis. This database holds significant potential in the realms of diagnosis, treatment, and prevention of EMT-related disorders, including cancer metastasis.

The supplementary information regarding sample details can be found in Supplementary Table 2.

The sample clinical information statistics of the TCGA HNSCC cohort are presented in Supplementary Table 3.

### Construction and survival analysis of prognostic risk model

The coxph function from the R package survivor was employed to conduct a one-way Cox regression analysis on genes within the key module, aiming to identify genes significantly associated with overall survival (*P* < 0.01). The outcomes of this analysis were visually represented using the forest plot feature of the R package. Additionally, the glmnet package in R was utilized to perform LASSO regression, enabling the identification of key prognosis-related genes. Subsequently, a multifactor regression prognostic model was constructed. The threshold point for classifying tumor samples into high- and low-risk populations was determined based on the median risk score. Prognostic analysis was conducted by generating survival curves using the Kaplan–Meier method, and significant differences were measured using the log-rank test. To evaluate the predictive ability of the perturbation scoring model, ROC curves were plotted using the R package time ROC. In this model, the risk value is calculated as the sum of each candidate gene expression value multiplied by its corresponding weight.

$$ {\text{Riskscore}} = \mathop \sum \limits_{i = 0}^{n} \beta i \times \chi i $$

A combined plot of survival and score was also plotted using the R package ggplot2, containing a scatter plot of survival time and survival status and a scatter plot of sample score.

### Immune cell infiltration ratio

The CIBERSORT algorithm is employed as a methodological approach to ascertain the cellular composition within intricate tissues, primarily relying on gene expression patterns^10^. By utilizing the leukocyte signature gene matrix LM22, encompassing a total of 547 genes, we were able to successfully identify and classify 22 distinct immune cell types, encompassing myeloid subpopulations, natural killer (NK) cells, plasma cells, naive and memory B cells, as well as seven distinct types of T cells. The IOBR cybersport algorithm, integrated within the R package, was employed to accurately compute the proportions of these aforementioned 22 cell phenotypes within the provided data, ensuring that the cumulative proportions of each immune cell type within every sample remained consistent.

### qRT-PCR

A total of twenty-eight samples of head and neck squamous cell carcinoma (HNSCC) and their corresponding adjacent non-cancerous tissue were procured from the Department of Oral and Maxillofacial Surgery at the First Hospital of Harbin Medical University. The research adhered to the guidelines outlined in the Declaration of Helsinki by the World Medical Association. Prior to inclusion in the study, informed consent was obtained from all participating patients, and approval from the hospital ethics committee was obtained. Furthermore, none of the specimens had undergone any form of preoperative radiotherapy or chemotherapy. HNSCC and matched adjacent non-cancerous mucosa (taken from at least 5 cm from the tumor margin) were collected and immediately frozen in liquid nitrogen before being stored at -80 °C until subsequent RNA extraction.

The tissue samples were subjected to mRNA extraction using the TRIzol reagent under low temperature conditions. The resulting mRNA was subsequently diluted based on concentration and subjected to reverse transcription using a reverse transcription kit. The thermal cycling conditions for RNA quantification involved a series of cycles consisting of 95 °C for 10 min, followed by 95 °C for 15 s, 55 °C for 30 s, and 72 C for 30 s, repeated for a total of 40 cycles. The melting phases included steps at 95 °C for 15 s, 60 °C for 1 min, and 95 °C for 15 s. The experiments were repeated three times independently, and melting curve analysis was conducted on each sample to confirm the specificity of amplification. The primer sequences for the genes can be found in Supplementary Table 4.

### Significance labeling notes

The Wilcoxon test was employed to assess disparities between two sets of samples, while the Kruskal–Wallis test was utilized to evaluate distinctions among multiple groups of samples. The notation "ns" indicates a *P*-value greater than 0.05, "*" denotes a *P*-value of 0.05, "**" denotes a *P*-value of 0.01, "***" denotes a *P*-value of 0.001, and "****" denotes a *P*-value of 0.0001.

## Results

### Glycosylation-Related Gene Set

**Figure 1:**
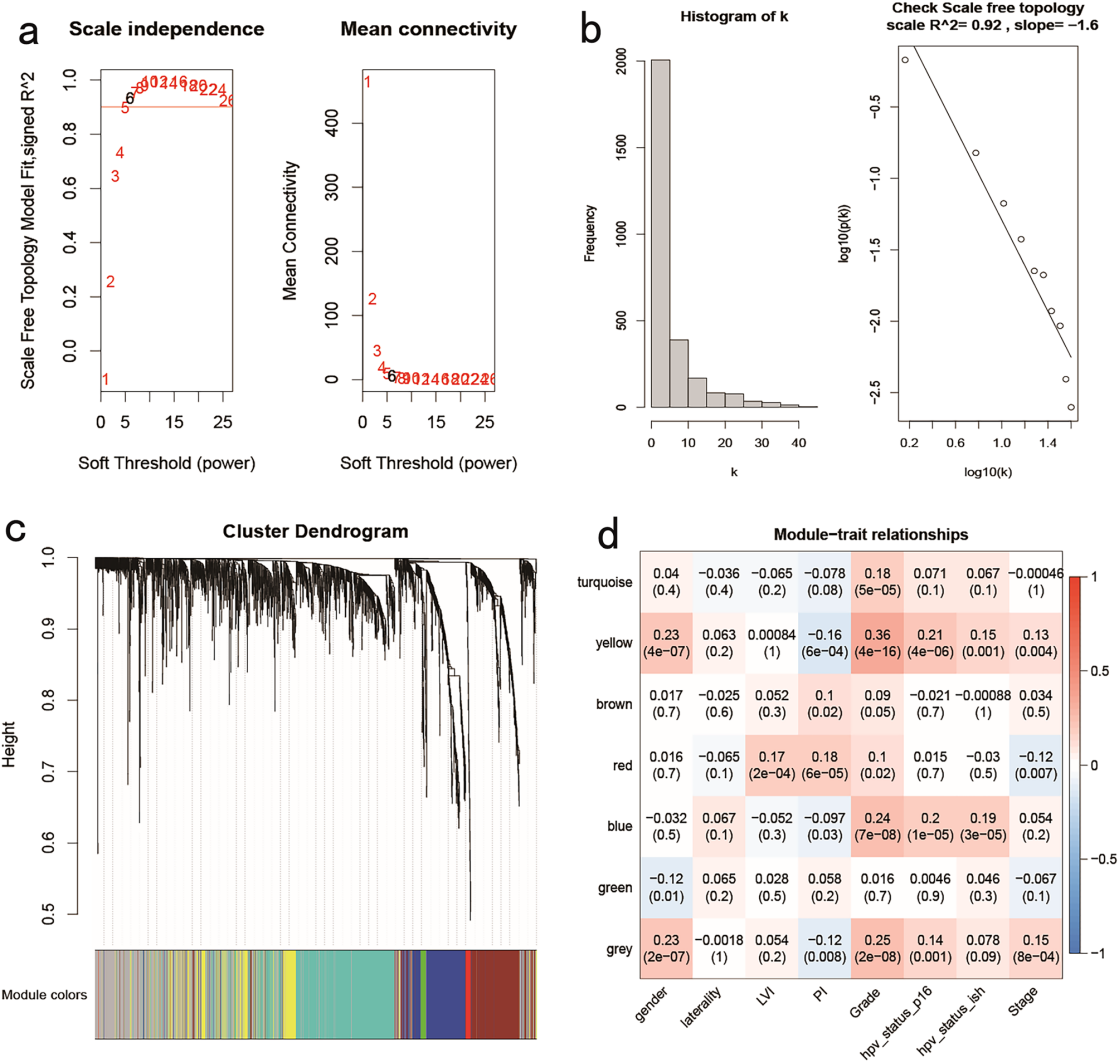
Results of weighted gene co-expression network analysis. (**a**, **b**) Scale independence and average connectivity among soft thresholds β. (**c**) Cluster tree plot of module genes. (**d**) Heat map of module trait correlations. Each row is a color module, and each column is a clinical characteristic. Each cell includes module correlation coefficients, clinical traits, and corresponding P-values in parentheses.

The initial HNSC cohort, which included outlier samples, was excluded, resulting in a total of 487 samples for further analysis. Co-expression networks were constructed using a correlation coefficient threshold of 0.9, a minimum module size of 30 genes, a maximum module distance of 0.3, and Pearson correlation as the calculation method for both co-expression correlation and module trait correlation. The findings are presented in Fig. 1A, where a correlation coefficient exceeding 0.9 leads to the determination of an optimal soft threshold of 6. Figure 1B demonstrates a negative correlation between k and p(k) (correlation coefficient = 0.92), suggesting that the chosen β value can be employed for constructing a gene scale-free network. Figure 1C exhibits the module clustering tree, with the upper section representing the gene clustering tree and the lower section depicting the clustering tree based on Similarity clustering into modules. Figure 1D illustrates that the yellow module exhibits the highest correlation with the Grade trait (correlation coefficient = 0.36), followed by the gender trait (correlation coefficient = 0.23). Similarly, the blue module also displays a notable correlation with the Grade trait (correlation coefficient = 0.24). Consequently, the pivotal modules in this analysis are identified as yellow and blue, while the crucial traits are determined to be Grade and gender.

The set of key glycosylation factor genes associated with the disease was determined by establishing the genes of two key modules. Subsequently, a total of 749 key glycosylation genes were subjected to one-way Cox regression analysis to identify genes associated with overall patient survival (OS). This analysis yielded 38 glycosylation factors that exhibited significant associations with prognosis. The results of the univariate analysis were presented using forest plots, with the median expression serving as the cutoff point (Supplementary Table 1). The *P*-values of the hypothesis test assessing the survival prognosis of the genes were all found to be less than 0.05, indicating a statistically significant difference in prognosis between the subgroups (Supplementary Fig. 2).

**Figure 2:**
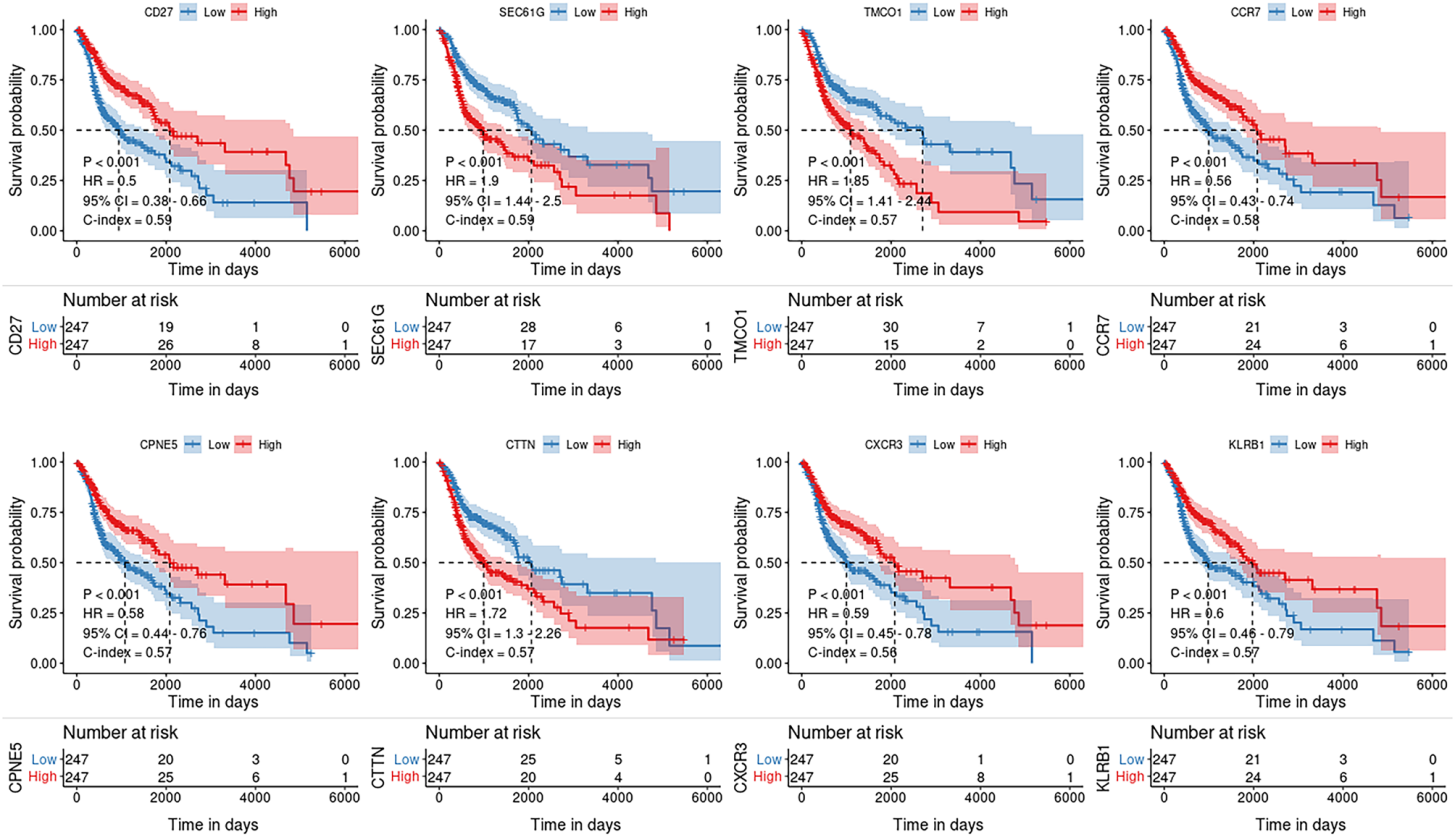
Kaplan Meier survival curves for eight genes. CD27, SEC61G, TMCO1, CCR7, CPNE5, CTTN, CXCR3, and KLRB1, were selected to draw KM curves. The differences between the high and low groups were significant (*p* < 0.001).

Kaplan–Meier (KM) survival curves were generated using the median gene expression as the cutoff value for distinguishing the high and low subgroups. Among these subgroups, the top eight genes (CD27, SEC61G, TMCO1, CCR7, CPNES, CTTN, CXCR3, and KLRB1) with the most pronounced prognostic disparities were identified. For the categories at high- and low-risk for the eight genes, *P* < 0.001 indicates a significant difference in prognosis (Fig. 2).

### Correlation of glycosylation risk score (GLRS)

Using the LASSO linear regression method, a total of 16 prognostic-related marker genes (F5, OLR1, AREG, CTLA4, CLEC2D, JCHAIN, NINJ1, CD6, CCR7, CTTN, ADAMTS1, SMS, PKD1, MASP1, TMCO1, and TPSAB1) were selected from a pool of 38 prognostic glycosylation-related genes. These marker genes were then utilized to construct the glycosylation risk score (GLRS), with redundant genes eliminated (Supplementary Fig. 3).

**Figure 3:**
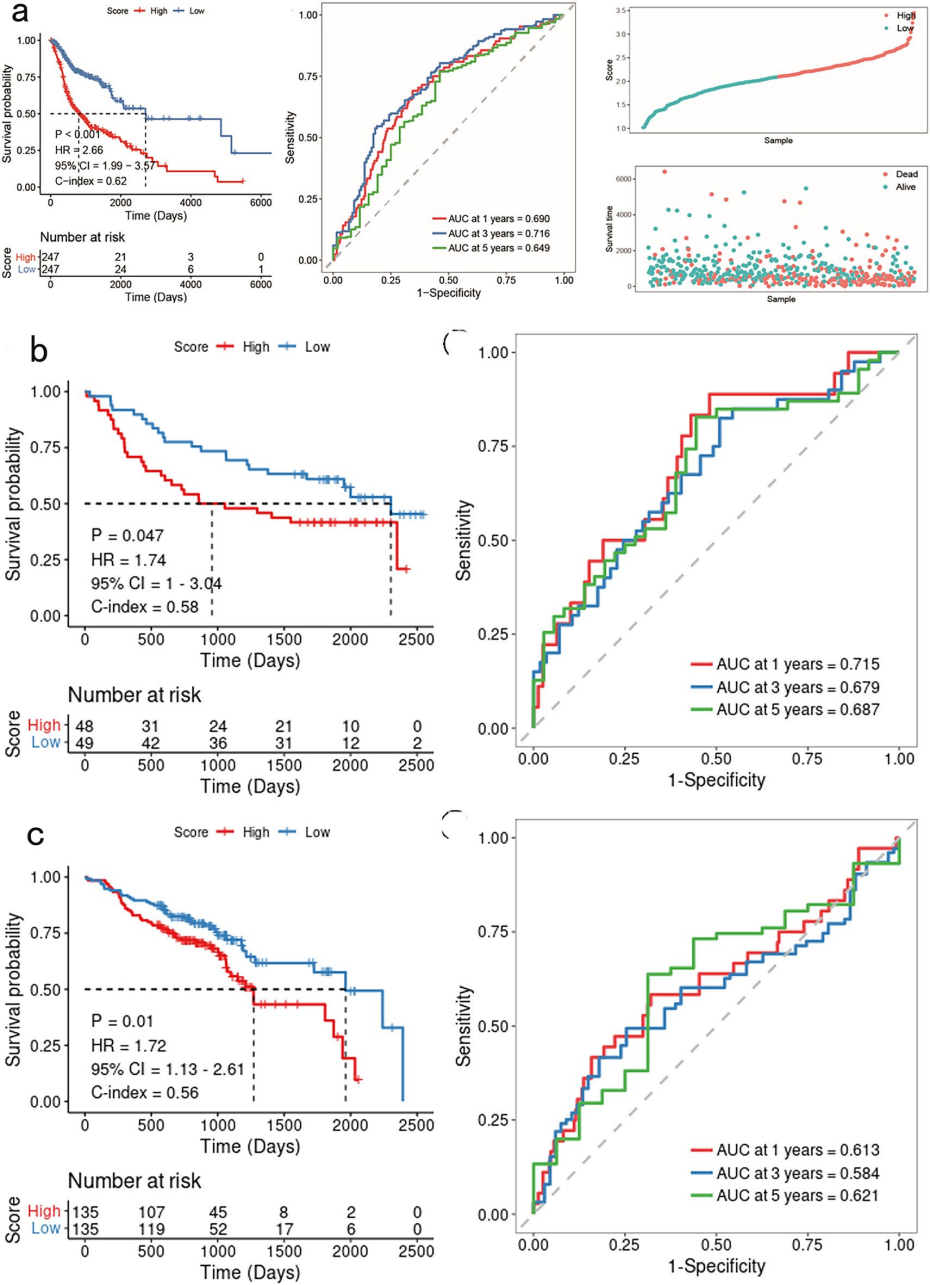
Results of validating model robustness on training and validation sets. (**a**): Training set KM, ROC curve, and scatter plot of survival state and survival time. (**b**) GSE41613 validation set: KM and ROC curves. (**c**) GSE65858 validation set: KM and ROC curves.

The TCGA HNSCC dataset samples were categorized as high or low risk according to the median value of their risk scores, which was considered the pivotal threshold. Additionally, Kaplan–Meier (KM) curves were generated to evaluate the prognostic disparities between these two groups. The findings demonstrated that the high-risk group exhibited an unfavorable prognosis, and the KM curves comparing the high- and low-risk groups (*P* < 0.001) indicated a significant divergence in their prognoses (Fig. 3a).

The predictive validity of the glycosylation risk score, which comprised 16 genes, in forecasting the overall survival of patients with HNSCC at 1, 3, and 5 years was further confirmed through the utilization of receiver operating characteristic (ROC) analysis. The results demonstrated that the AUC values for the respective time points were 0.690, 0.716, and 0.649 (Fig. 3a).

Scatter plots were constructed to depict the correlation between survival time and survival status, as well as the association between sample risk scores and survival. By merging these scatter plots, the analysis revealed a negative relationship between the risk score and the duration of patient survival, indicating that higher risk scores were associated with shorter survival times (Fig. 3a).

In the validation sets GSE41613 and GSE65858, the samples were stratified into high- and low-risk groups based on the median value of the sample's risk score. Subsequently, Kaplan–Meier (KM) and receiver operating characteristic (ROC) curves were constructed. The results from both validation sets revealed that the high-risk group exhibited a poorer prognosis. The KM curves demonstrated a statistically significant difference in prognosis between the high- and low-risk groups (*P* < 0.001). Furthermore, the projected area under the curve (AUC) values at 1, 3, and 5 years were higher, indicating a favorable validation of the model (Figs. 3b,c).

### GLRS with clinical characteristics

**Figure 4:**
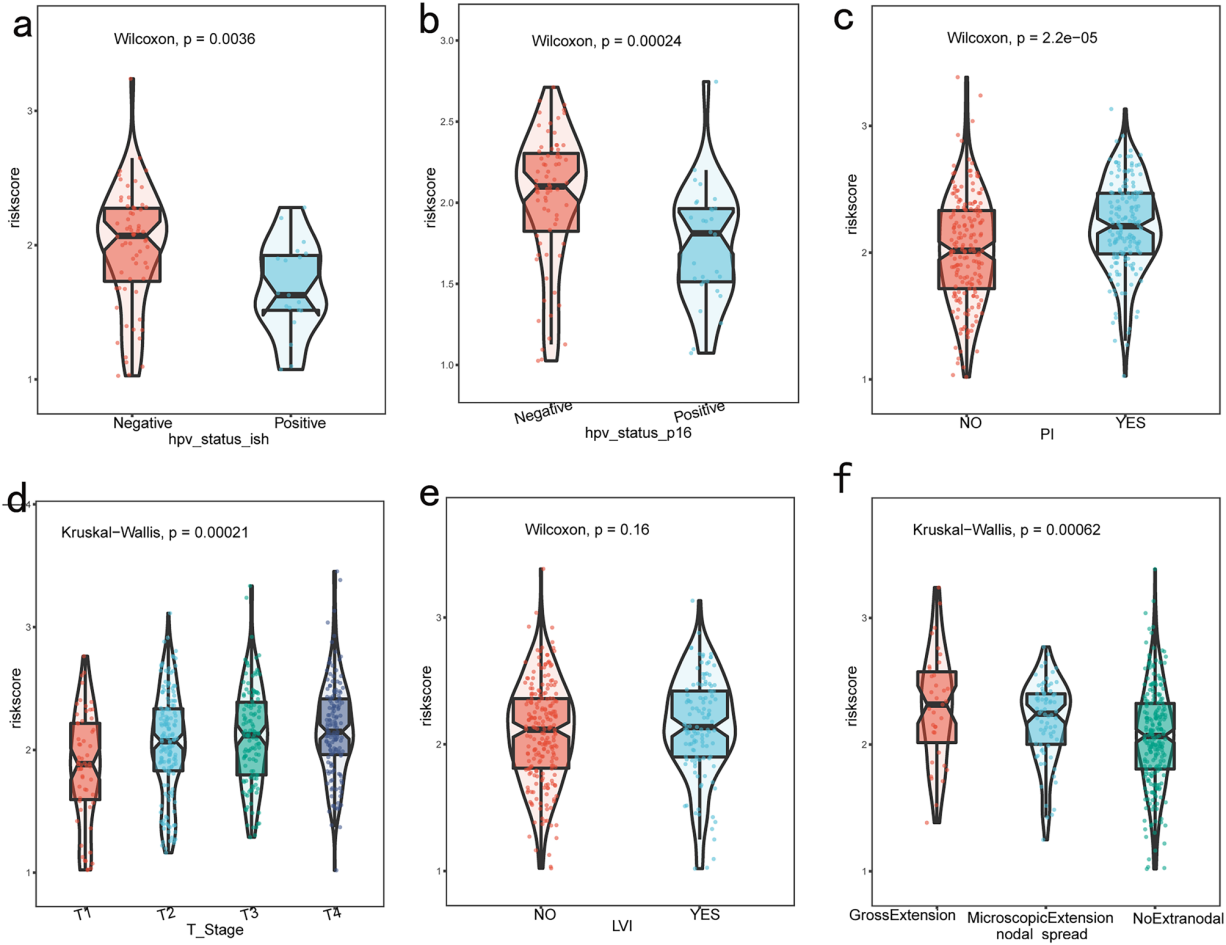
Clinical characteristics and box plot of GLRS score. (**a**, **b**) Box plot of differences in Hpv infection status scores. (**c**) Box plot of scoring differences in peripheral nerve infiltration. (**d**) Box plot of differences in lymph node diffusion status scores. (**e**) Box plot of scoring differences in cancer cell infiltration status. (**f**) Box plot of differences in ratings of surrounding aggression states.

The study investigated the correlation between various clinical characteristics and model scores by examining the groups of clinical characteristics and the risk scores of each sample in the model. Notably, significant disparities were observed in the risk scores of clinical characteristics when categorized by lymph node spread status, perineural infiltration, HPV infection status, and T stage (Fig. 4).

Univariate and multifactor Cox regression analyses were conducted to assess the potential of GLRS as an independent prognostic factor, in the absence of other clinical variables. The validity of this analysis was confirmed by incorporating age, tumor stage, gender, lymphovascular invasion, and peripheral nerve invasion in the HNSCC samples. The results indicated a strong association between patients' overall survival time and the univariate Cox regression analysis, as well as lymphovascular invasion, peripheral nerve invasion, and GLRS (Supplementary Fig. 4). After conducting additional adjustments using multifactorial Cox regression analysis, it was observed that GLRS exhibited a significant association with the overall survival time of patients with HNSCC (*P* < 0.001, 95% CI 0.28–0.50, HR = 0.38). These findings indicate that GLRS has the potential to independently predict patient prognosis, irrespective of clinically relevant variables, thereby suggesting its utility as an independent prognostic indicator for HNSCC patients.

**Figure 5:**
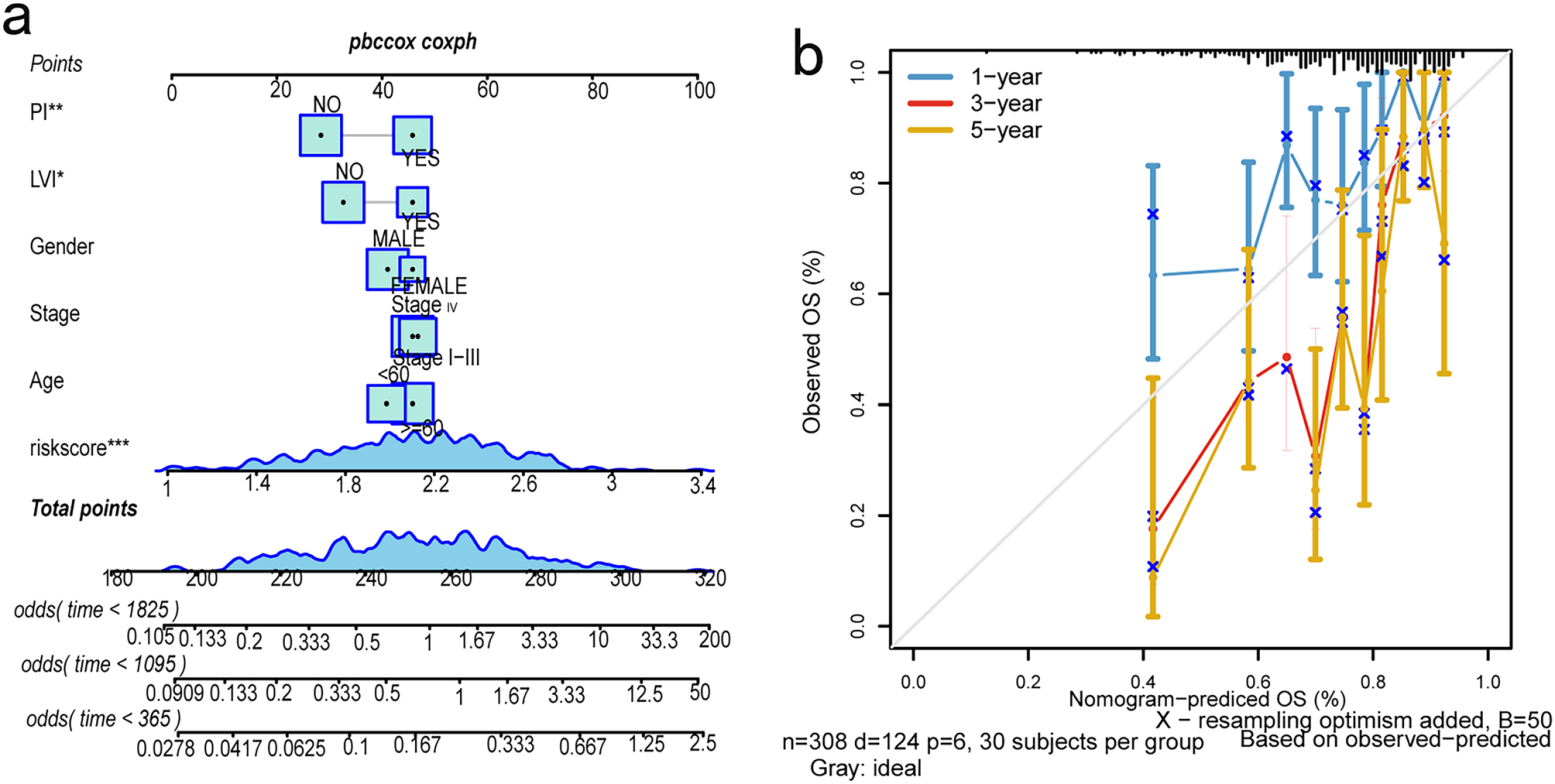
Multiple regression column chart and calibration plot. (**a**) Column chart constructed by combining GLRS risk score with survival time, survival status, and clinical variables. (**b**) Calibration curves for overall survival (OS) of patients at 1, 3, and 5 years.

Furthermore, a column chart (Fig. 5a) is constructed by incorporating survival time, survival status, clinical variables, and risk scores. Each variable's corresponding value is aggregated to form the column chart. The cumulative points on the lower scale of the chart correspond to the estimated likelihood of 1-year, 3-years, and 5-years overall survival (OS). The findings demonstrate that the GLRS risk score exerts the most significant influence on survival prognostication, implying its commendable efficacy in predicting the overall survival rate.

The utilization of a calibration plot (Fig. 5b) serves to visually assess the efficacy of the column chart, with the 45-degree line serving as the benchmark for optimal prediction. This plot effectively exhibits the calibration curves for the prediction of 1-year, 3-year, and 5-year overall survival (OS) in patients with head and neck squamous cell carcinoma (HNSCC). The findings indicate a satisfactory performance of the column chart.

The tumor microenvironment is a product of the dynamic interplay between various cellular and non-cellular components. It is not a solitary entity, but rather a collective of performers, including cancer cells, fibroblasts, myofibroblasts, endothelial cells, and immune cells. This brief review emphasizes the significant immune infiltrates present within the tumor microenvironment that influence the development of CTL-rich immune hot and CTL-deficient immune cold tumors, as well as novel strategies that may enhance our immune responses in both types of tumors.

### Validating core genes

**Figure 6:**
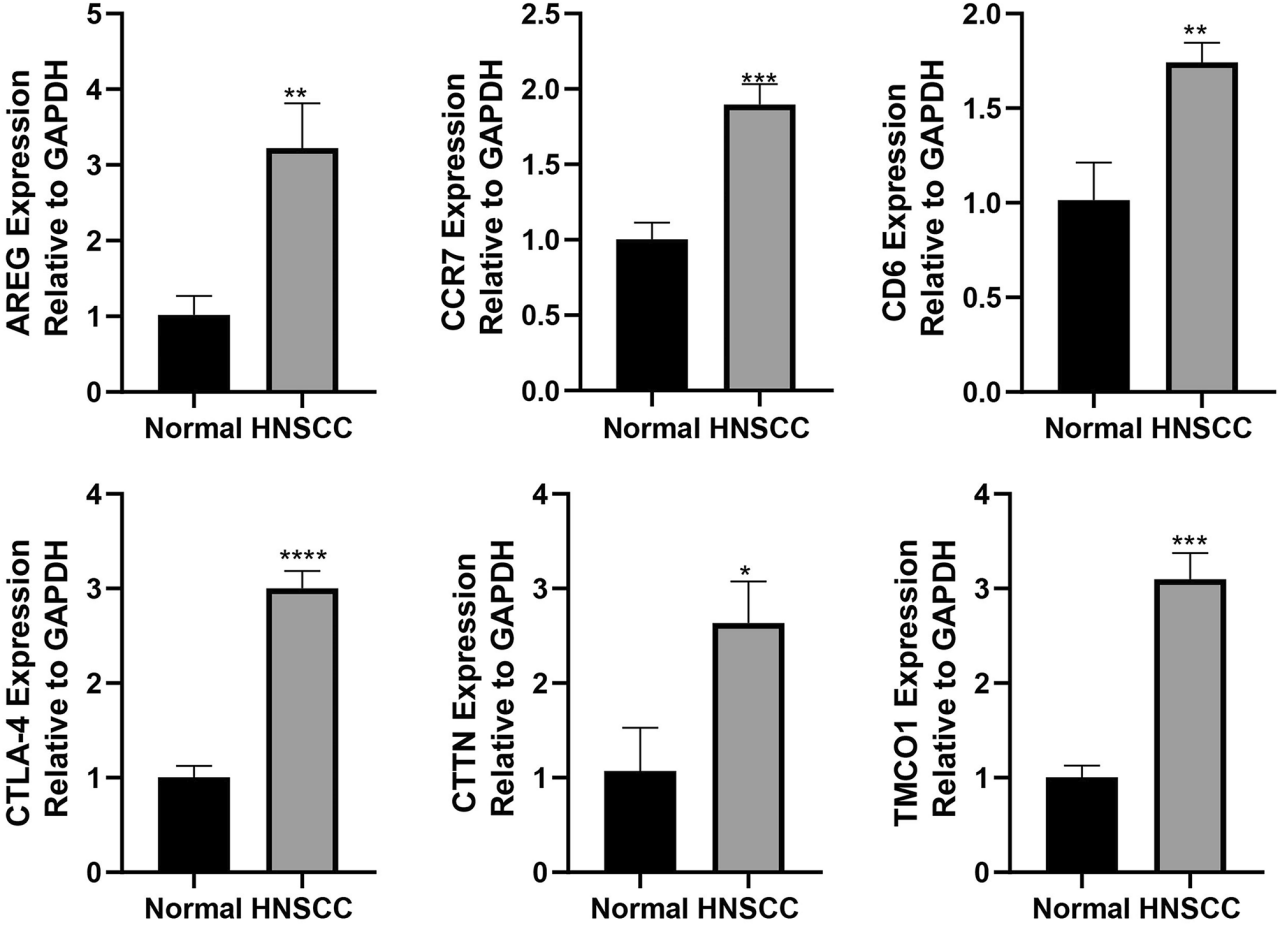
Validation of the expression of AREG, CCR7, CD6, CTLA-4, CTTN, and TMCO-1 using qRT-PCR.

To enhance the credibility of the core genes, a subset of six genes (AREG, CTLA4, CD6, CCR7, CTTN, and TMCO1) was chosen for analysis of their mRNA expression levels in 28 pairs of genomes. The differential expression of these genes between cancerous and adjacent non-cancerous tissues was determined using qRT-PCR. The objective of this study was to investigate the disparities in gene expression between cancerous tissue and neighboring non-cancerous tissue using quantitative real-time polymerase chain reaction (qRT-PCR). The findings revealed that all six genes exhibited upregulation in the tumor tissue (Fig. 6), which was by the database analysis outcomes conducted within this research. These results provide further validation for the potential utilization of these genes as biomarkers for HNSCC.

CD6, a transmembrane glycoprotein with a molecular weight ranging from 105 to 130 kDa, is predominantly expressed by lymphocytes, encompassing mature T cells and approximately 50% of NK cells^11^. Investigations have been conducted to assess the impact of the anti-CD6 monoclonal antibody UMCD6 on the cytotoxicity of human lymphocytes against cancer cells. The findings indicate that UMCD6 augments the cytotoxicity towards breast cancer, lung cancer, and prostate cancer cells by directly influencing CD8 T cells and NK cells. In comparison to the monoclonal antibody checkpoint inhibitor that disrupts the programmed cell death 1 (PD-1)/PD-1 ligand 1 (PD-L1) axis, UMCD6 exhibits greater efficacy in inducing cancer cell death and decreasing the survival rate of cancer cells in vitro^12^. Consequently, CD6 emerges as a prospective target for cancer immunotherapy. The protein TMCO1, which consists of transmembrane and coiled helical domains, has been identified as a highly conserved Ca^2+^ channel protein. Recent research has revealed that TMCO1 plays a crucial role in maintaining Ca^2+^ balance by preventing excessive Ca^2+^ accumulation and facilitating the release of Ca^2+^ from the endoplasmic reticulum into the cytosol^13^. On the other hand, the role of oncogenes and tumor suppressors in regulating Ca^2+^ in vivo balance has attracted increasing attention^14^. CC chemokine receptor 7 (CCR7) is a well-established receptor found on immune cells that facilitates their migration to lymph nodes. It has been extensively studied and identified as a crucial molecule in the process of lymph node metastasis^15^. Researchers hypothesize that the interaction between CCR7 and its corresponding ligand, CC chemokine ligand 21/secondary lymphoid tissue chemokine (CCL21/SLC), may be responsible for the occurrence of lymph node metastasis. In previous studies, Cunningham et al. and Wiley et al. have documented the significant metastatic capacity of CCR7-expressing cells in breast cancer and malignant melanoma metastasis^16,17^. The recent advancements in targeting immune checkpoint molecules, including PD-1, PD-L1, and CTLA4, have indicated that the suppression of tumor-specific immunity plays a crucial role in the pathology of HNSCC^18^. The prognostic outcomes of T cell infiltration and characteristics are often interconnected. The process of T cell transformation into an immunosuppressive state is facilitated by the presence of tox, PDCD1, CTLA4, and CD28, suggesting that the immunosuppressive potential of T cells can be hindered by inhibiting these molecules. The ability to unleash T cells holds significant importance in the realm of cancer immunotherapy^19^. Over-expression of CTTN has been observed, which is a significant factor in the development of head and neck tumors, particularly in the progression of lymph node metastasis^20^. Cortactin, a protein encoded by the CTTN gene located in chromosome band 11q13^21^, is frequently amplified at this chromosomal locus, leading to the overexpression of cortactin^21^. This overexpression of cortactin is a crucial event in the formation of head and neck cancers^22^. Furthermore, CTTN overexpression has been widely documented in esophageal tumors, which promotes carcinogen-induced cell migration as well as resistance to anorexia^23^. Many studies have shown that soluble media from the microenvironment can promote the growth of cancer and treatment resistance^24,25^. Amphibulin (AREG) is a fully processed secretion resulting from a sequence of protein hydrolysis events after the synthesis of transmembrane precursors^26^. Numerous studies have indicated that AREG is implicated in drug resistance across various cancer cell types, such as breast cancer, liver cancer, pancreatic cancer, and colorectal cancer^27,28^.

### Correlation of GLRS with the tumor microenvironment

**Figure 7:**
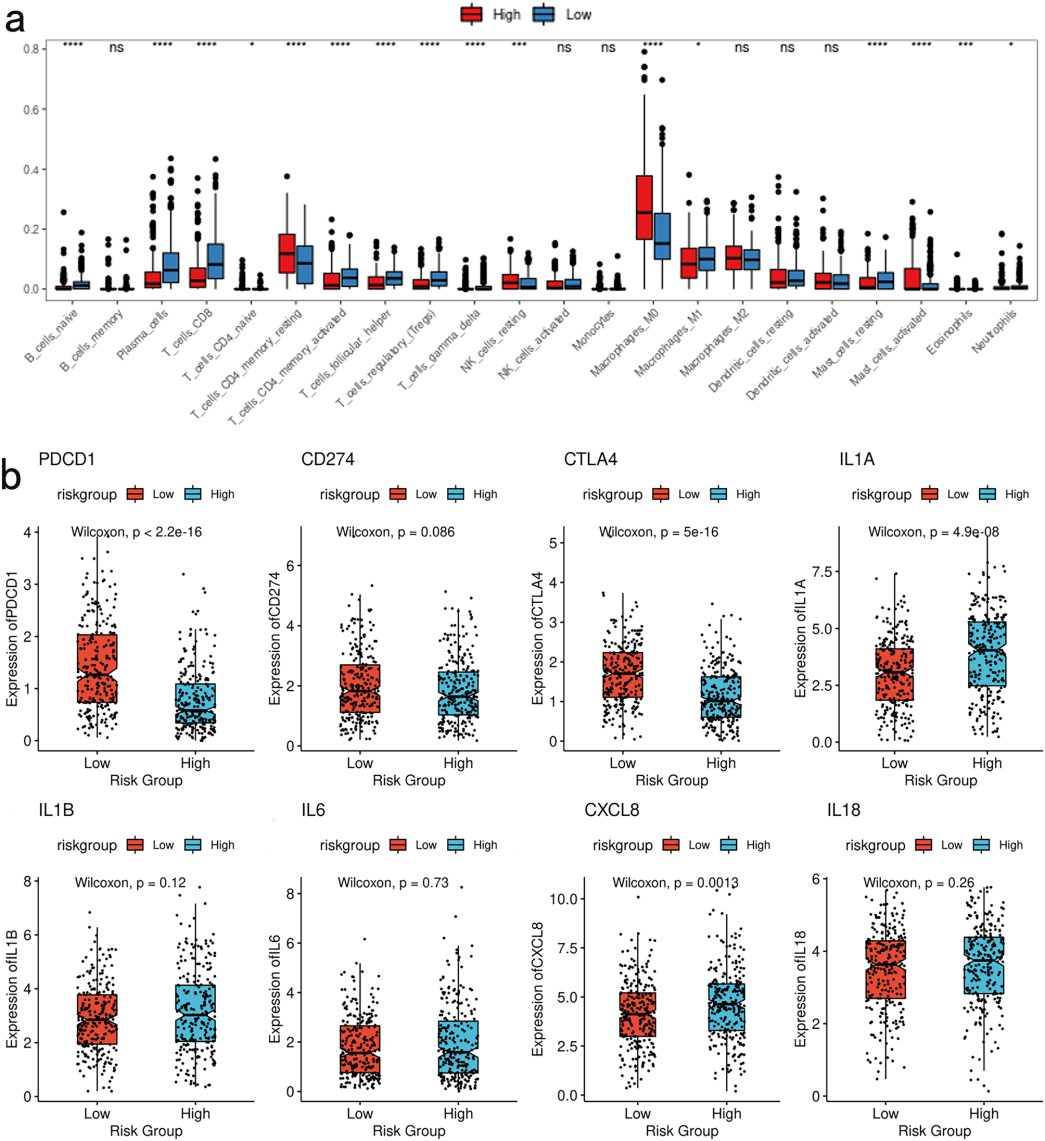
GLRS and immune-related outcomes. (**a**) Differences in immune infiltration between high and low-risk groups. (**b**) Differences in expression of immune checkpoint between high and low-risk groups.

Using the TCGA-HNSC dataset, the proportions of 22 immune cell infiltrates were computed for the low- and high-risk subgroups. P-values were determined through the application of a rank-sum test (Wilcoxon), while box plots were employed to visually represent the variations in immune infiltration between the aforementioned groups (Fig. 7a). The majority of immune cell infiltrates exhibited statistically significant disparities.

Immune checkpoints, which are molecules generated by immunological cells, play a critical role in modulating immune activation and are pivotal in the development of autoimmune manifestations in humans. As depicted in Fig. 7b, notable variations in the expression of prominent immune checkpoints, namely PDCD1, CTLA4, IL1A, and CXCL8, were observed between high- and low-risk subgroups. These differences were statistically significant in both high- and low-risk groups, highlighting the substantial disparities in the expression levels of PDCD1, CTLA4, IL1A, and CXCL8.

### GLRS embodiment in biology

**Figure 8:**
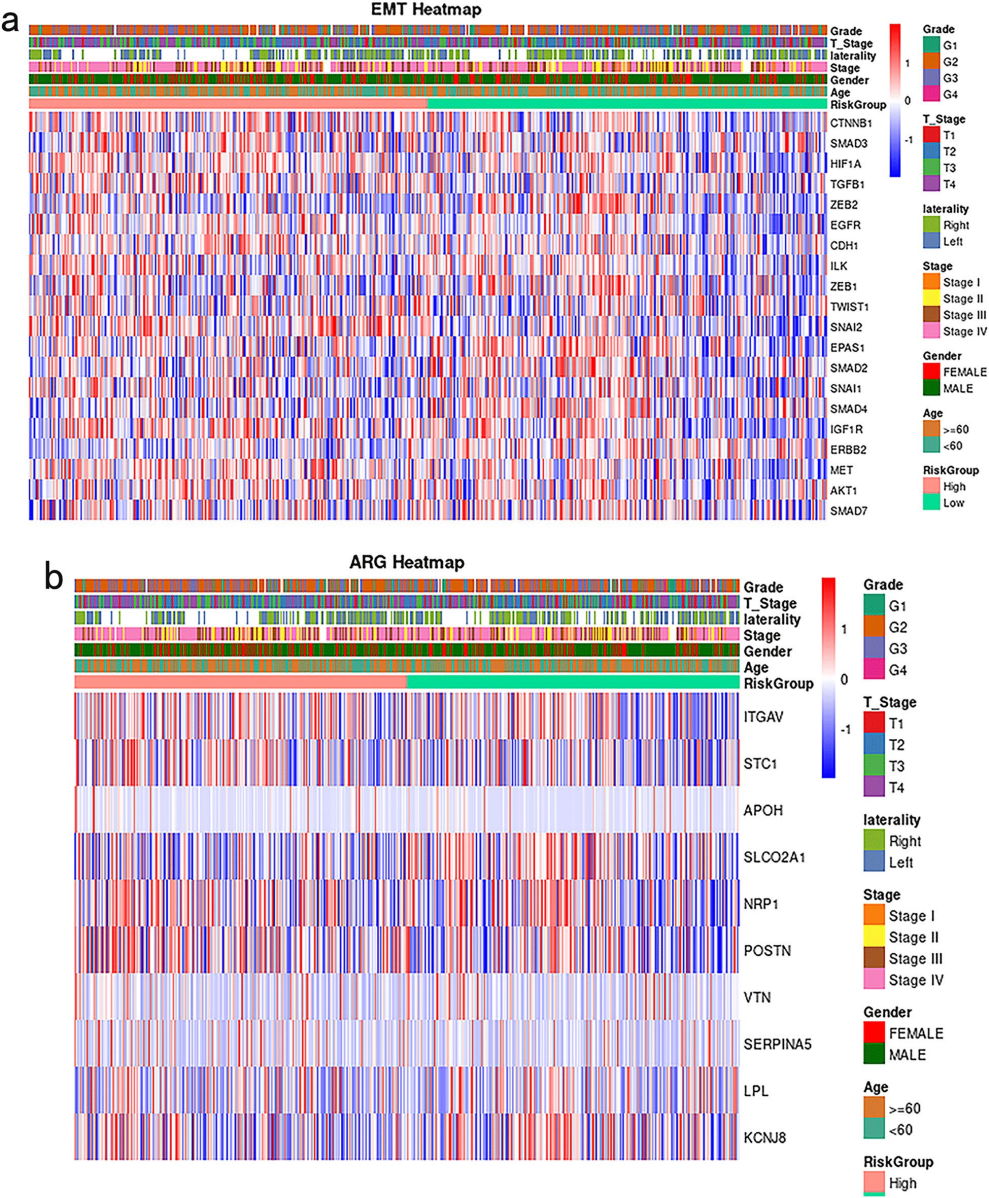
Differential expression of GLRS in biological factors. (**a**) Heat map of EMT gene expression. (**b**) Heat map of angiogenic factor expression.

Epithelial to mesenchymal cell transformation (EMT) is a cellular process that holds substantial importance in the progression of tumors and their metastasis. In order to illustrate the variations in gene expression among high- and low-risk subgroups compared to subgroups based on clinical characteristics, a heat mapping approach was employed, focusing on the top 20 genes closely linked to the EMT process (Fig. 8a). The results showed that the TOP20 EMT gene had significant expression differences between high and low groups.

The dysregulation of glycosylation regulation of VEGFR, which interacts with galectins, has been found to be linked with tumor angiogenesis. In order to illustrate the variations in the expression of angiogenic factors among high- and low-risk categories as well as clinical characteristics subgroups, a heat map (Fig. 8b) was employed. The results showed that there were significant differences in the expression of ITGAV, KCNJ8, NRP1, POSTN, SERPINA5, STC1, and VTN between the high and low-risk groups.

## Discussion

Despite the presence of various clinical symptoms and the availability of diagnostic methods through relevant clinical examination, a considerable number of individuals diagnosed with neck and head squamous cell cancers exhibit advanced lesions^29^. Numerous cancer biomarkers have been developed and utilized across various cancer types, such as carcinoembryonic antigens in lung and gastrointestinal cancers^30^, prostate-specific antigens in prostate cancer, and CA19-978 in pancreatic cancer^31,32^. However, the squamous cell carcinoma antigen stands as the sole viable biomarker for squamous cell carcinoma (SCC), which predominates in head and neck cancers^33^. The emergence of bioinformatics analysis algorithms has led to the development of numerous algorithms for disease prediction, including cancer markers. The progress in interaction prediction research across various domains of computational biology offers valuable insights into genetic and ncRNAs related markers associated with HNSCC, such as miRNA-lncRNA interaction prediction^16,34^, and metabolic markers^35,36^. In addition, some deep learning models also provide excellent cancer big data analysis tools^37–40^. Ordinary differential equation-based theoretical modeling studies on gene/protein signaling networks were important for understanding regulatory mechanisms and finding potential therapeutic targets in diseases^41–43^.

Glycosylation represents the most intricate post-translational modification of proteins, and aberrant glycosylation serves as a distinctive hallmark of cancer. Changes in glycans on the surfaces of tumor and host cells, as well as in the tumor microenvironment, have been recognized as pivotal occurrences that can contribute to the development and advancement of cancer^44,45^. Recent investigations have unveiled that abnormal glycosylation can result in the secretion of polysaccharides or glycoproteins associated with tumors, thereby enabling their release into the bloodstream as markers linked to tumors^46^. Altered glycosylation directly impacts tumor proliferation and viability, while also fostering immune regulation and metastasis induced by tumors^47^. Moreover, glycosylation exerts a significant influence on the regulation of tumor proliferation, invasion, and angiogenesis. Consequently, the investigation of the underlying mechanisms pertaining to tumor glycosylation and aberrant glycosylation, the identification of diagnostic markers associated with glycosylation, and the identification of precise therapeutic targets have emerged as prominent areas of interest in the field of biomedical research. The comprehension of the modifications in glycosylation patterns related to HNSCC is of paramount importance for the development of therapeutic strategies aimed at reinstating normal glycosylation profiles in cancerous cells.

Tumorigenesis, progression, and invasion are concomitant with alterations in glycosylation patterns of associated glycoproteins. The surface carbohydrate composition of cancer cells undergoes modifications throughout the course of the disease. Glycosylation processes exert significant regulatory effects on intricate protein functionality, thereby promoting tumor growth, invasion, and angiogenesis. In the context of HNSCC, abnormal glycosylation mediated by glycoproteins such as E-cadherin, PD-1/PD-L1, EGFR, and CD44, can exert a profound influence on immune evasion and epithelial-mesenchymal transition^48^. However, the mechanisms underlying the abnormal glycation of specific proteins in the development of HNSCC remain unknown.

In our study, we utilized Kaplan–Meier curves to assess the overall survival of patients with Head and Neck Squamous Cell Carcinoma (HNSCC). We observed that the inclusion of F5, OLR1, AREG, CTLA4, CLEC2D, JCHAIN, NINJ1, CD6, CCR7, CTTN, ADAMTS1, SMS, PKD1, MASP1, TMCO1, and TPSAB1 as glycosylation-related prognostic risk markers allowed us to construct the glycosylation risk score (GLRS). This GLRS can serve as a predictive biomarker for HNSCC, aiding in the prognosis of patients with this condition. The subsequent univariate and multifactorial Cox proportional risk regression analyses revealed a significant association between the risk score and overall survival (OS) of patients. This finding suggests that prognostic risk markers consisting of glycosylation-related genes have the potential to predict patient prognosis independently of clinical characteristics. Consequently, this study has successfully established glycosylation-related predictive risk factors as prognostic markers for patients with HNSCC, thereby offering novel perspectives and potential molecular targets for further investigation and targeted therapy in HNSCC. The results of our study suggest that prognostic risk indicators consisting of glycosylation-related factors have the potential to forecast prognosis and individualize treatment for patients with Head and Neck Squamous Cell Carcinoma (HNSCC). Additionally, this research offers novel insights into the investigation of tumor invasion and progression by examining the variations in expression levels of the top 20 genes strongly linked to the Epithelial-Mesenchymal Transition (EMT) process and angiogenic factors across different clinical characteristic subgroups within high- and low-risk subgroups.

The functionality of the innate and adaptive immune systems is reliant upon immune-related cells, including natural killer (NK) cells, dendritic cells (DC), B cells, and T cells. Throughout the process of carcinogenesis, abnormal glycosylation mechanisms possess the ability to directly or indirectly manipulate the immune response of tumor cells, consequently exerting control over tumor proliferation^49^. The examination of the impact of immunoglobulins, immune factors, and protein glycosylation on the morphology and functionality of immune cells holds potential for elucidating the influence of glycosylation on tumor immunity at a molecular level, as well as presenting novel therapeutic avenues for impeding tumor metastasis and recurrence. The precise mechanisms by which genes implicated in glycosylation modulate immune cell infiltration within the tumor immunological microenvironment remain elusive. This study examined the variations in expression of various immune checkpoints, commonly investigated, within high- and low-risk subgroups. Notably, the expression levels of PDCD1, CTLA4, IL1A, and CXCL8 exhibited significant disparities between the high- and low-risk subgroups. Additionally, the findings derived from the analysis of 16 glycosylation-related genes indicated a strong correlation between risk scores and the extent of infiltration by B cells, CD4 + T cells, CD8 + T cells, dendritic cells, and neutrophils.

Several variables influence prognoses, including the patient's psychological factors, tumor malignancy, the impact of early diagnosis and treatment, and age. Upon examining gene expression levels across different clinical phases, it was observed that not all genes exhibited an increase in expression with advancing tumor stage. In fact, certain genes demonstrated a decrease in expression during advanced tumor stages, suggesting their potential involvement in various clinical stages of tumorigenesis. TGF-β is commonly employed in the treatment of tumors due to its ability to impede cell growth and trigger apoptosis. Conversely, in advanced malignancies, TGF-β can act as a tumor promoter by inducing EMT, thereby initiating tumor promotion^50^. Prior investigations have revealed that CXCL10 exhibits both tumor-promoting and tumor-suppressing effects^51^. To facilitate future research on the mechanisms involved, it is imperative to explore the interplay between tumor grade, histological type, tumor stage, survival rates, and the regulatory network governing these genes. Notwithstanding the aforementioned investigations, the current study possesses certain limitations. Firstly, our study relied on data and survival information of HNSCC from the database, yet its limitations lie in the small sample size. Secondly, the study cohort we used was retrospective. The sufficient experimental verification and potential mechanism of GLRS in HNSCC still need further exploration. Thirdly, the prognosis lacks a comprehensive analysis of patient-specific characteristics such as age, gender, ethnicity, smoking and alcohol usage history, tumor stage, and grading. With the development of medical multi omics big data, more data with rich clinical samples and more suitable bioinformatics algorithms will greatly enhance our work.

### Supplementary Information

Supplementary Information.

## Data Availability

The facts and statistics in this article were all gathered from sources that were available to the public, including the following: TCGA (https://portal.gdc.cancer.gov/repository). GeneCards (https://www.genecards.org). GEO (http://www.ncbi.nlm.nih.gov/geo/).
