# Supplementary material for: Construction of an abnormal glycosylation risk model and its application in predicting the prognosis of patients with head and neck cancer
Source: Sci Rep. 2024 Jan 15;14:1310. doi: 10.1038/s41598-023-50092-6 (PMC10789784; doi:10.1038/s41598-023-50092-6)
Supplement: Supplementary file 1 — Supplementary Information. [file 41598_2023_50092_MOESM1_ESM.docx]

Supplementary Material

**Construction of an abnormal glycosylation risk model and its application in predicting the prognosis of patients with head and neck cancer**

Yihan Gao †^1,2^, Wenjing Li†^3^, Haobing Guo ^1,2^, Yacui Hao ^1,2^, Lili Lu ^1,2^, Songlin Piao ^1,2*^, and Jichen Li ^1,2*^

^1^Department of Oral and Maxillofacial Surgery, the First Affiliated Hospital of Harbin Medical University, Harbin/150000, China

^2^School of Stomatology, Harbin Medical University, Harbin/150000, China

^3^College of Animal Science, Zhejiang University, Hangzhou 310058, China

Yihan Gao†, Wenjing Li† These authors contributed equally to this work and share first authorship

*** Correspondence:**Songlin Piao; [songlinpiao@126.com](mailto:songlinpiao@126.com) and Jichen Li; lijichen@163.com.

Supplementary Figures


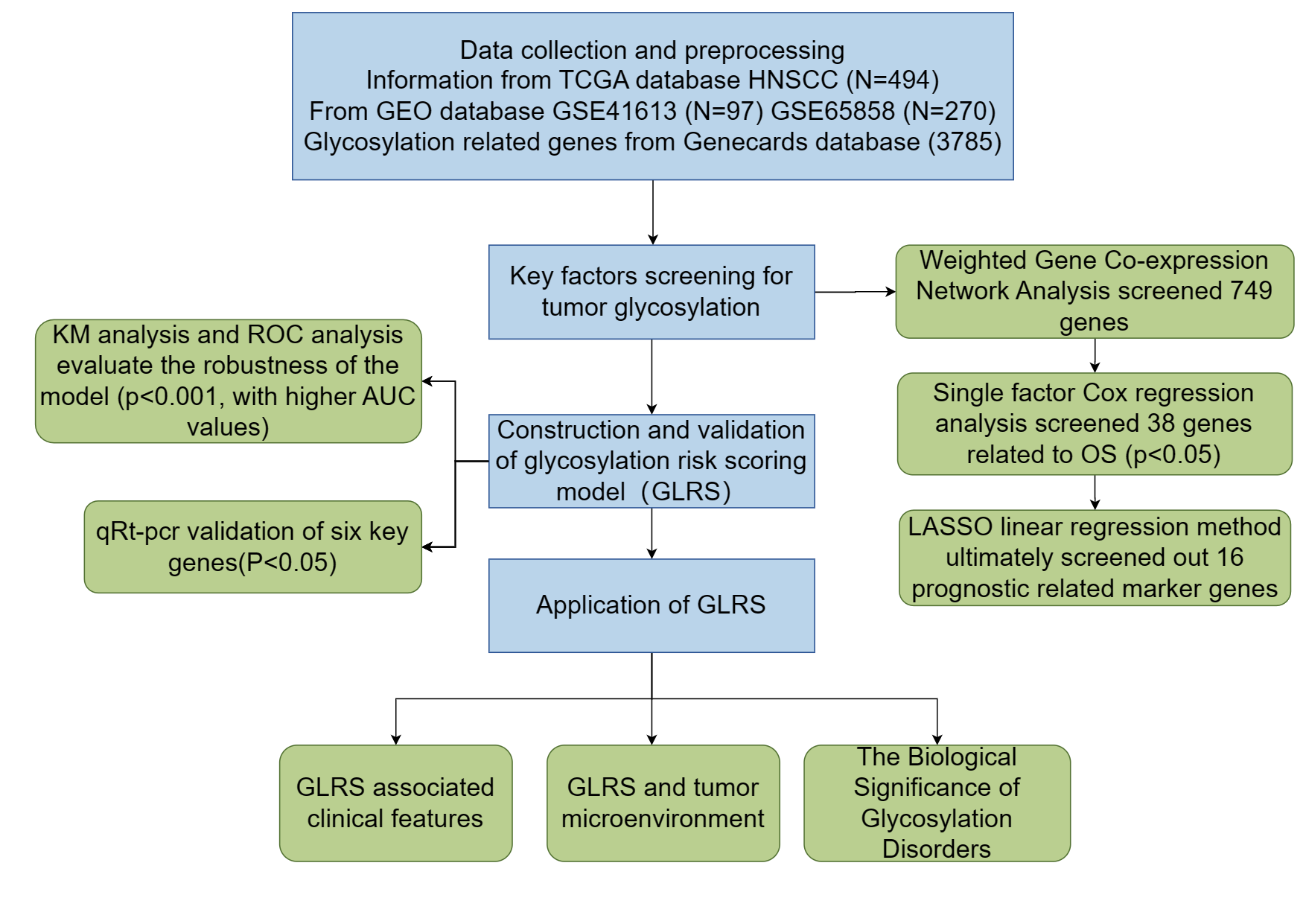


**Supplementary Figure1. Research flowchart**


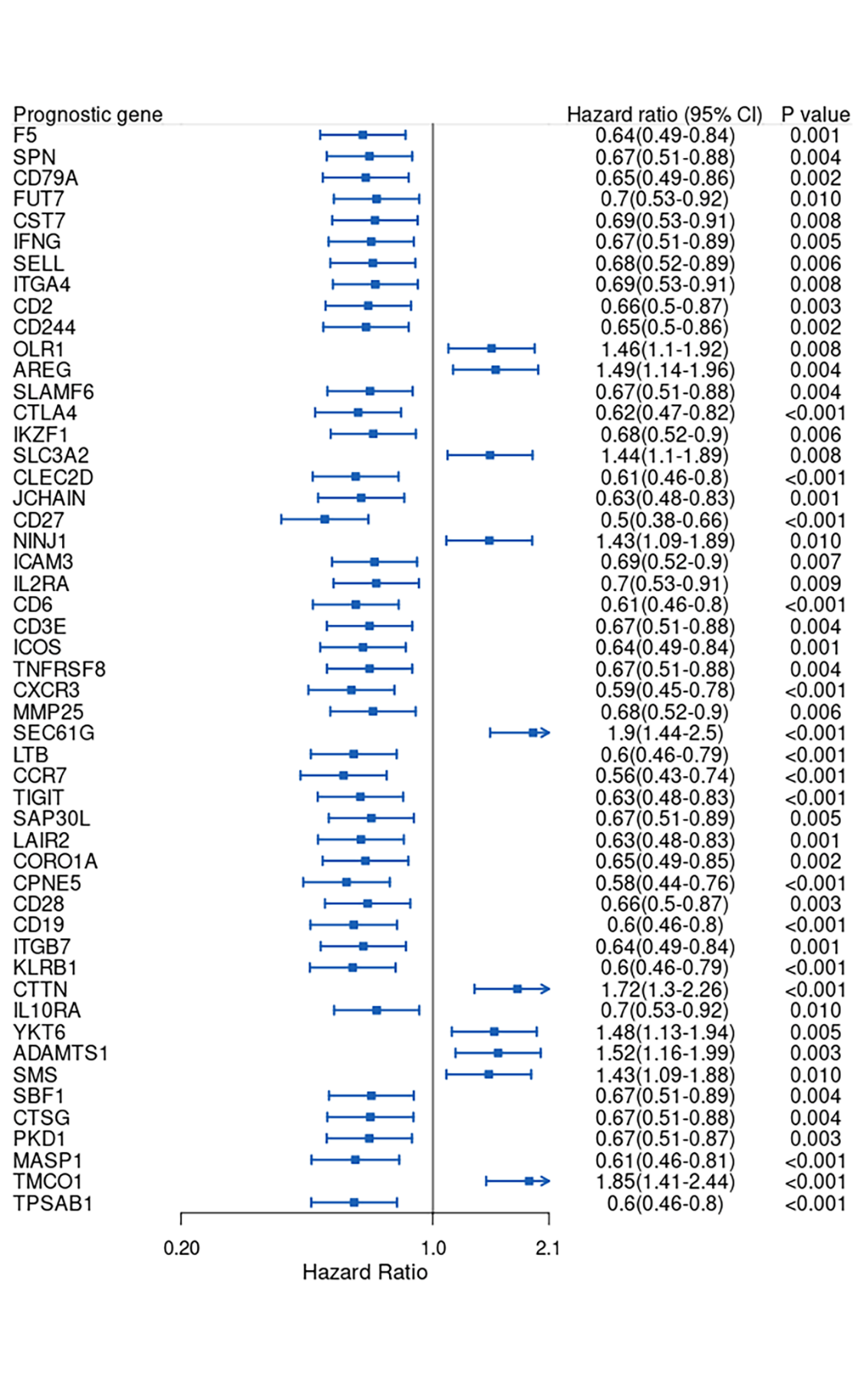


**Supplementary Figure2.** **Forest plot of prognostic factors** (squares on the horizontal line indicate hazard ratios; horizontal lines indicate 95% confidence intervals (CI))


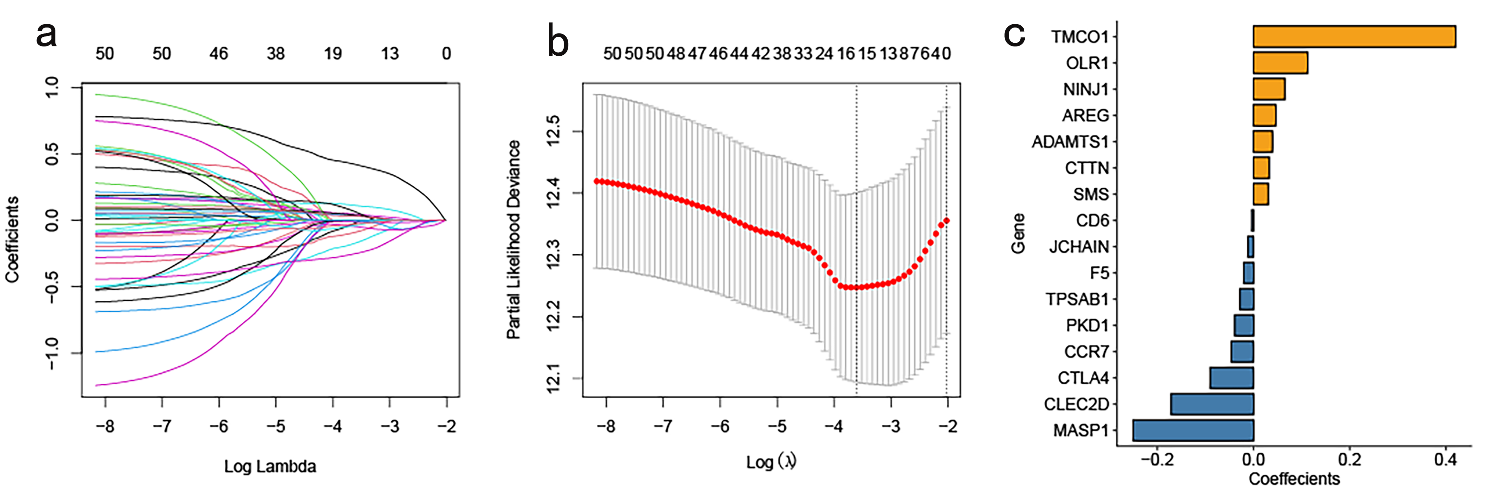


**Supplementary Figure 3.** **Lasso intermediate results diagram** **(a)** Confidence intervals for each Lambda in LASSO regression. **(b)**Trajectory of variable changes in LASSO regression, where the horizontal axis represents the logarithm of the variable Lambda, and the vertical axis represents the coefficients of the variables. **(c)** Coefficients of key prognostic genes in LASSO regression.


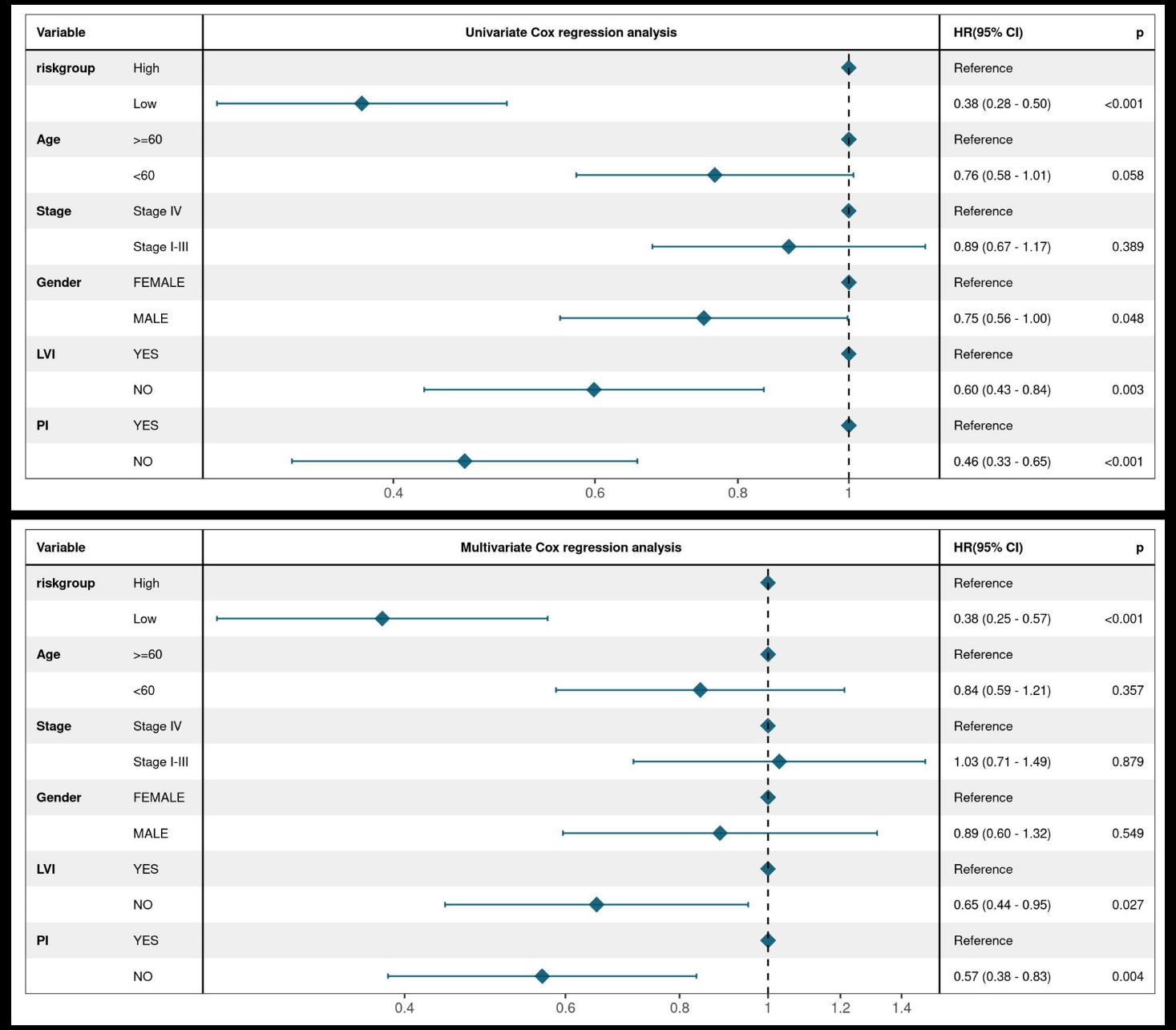


**Supplementary Figure 4. Single and multiple factor regression analysis between training set model grouping and some clinical indicators**

Supplementary Tables

| gene | Cutoff |
| --- | --- |
| F5 | 0.226 |
| SPN | 0.977 |
| CD79A | 2.615 |
| FUT7 | 0.311 |
| CST7 | 5.164 |
| IFNG | 0.265 |
| SELL | 2.166 |
| ITGA4 | 0.779 |
| CD2 | 5.343 |
| CD244 | 0.297 |
| OLR1 | 1.231 |
| AREG | 25.444 |
| SLAMF6 | 0.828 |
| CTLA4 | 1.524 |
| IKZF1 | 0.83 |
| SLC3A2 | 71.806 |
| CLEC2D | 0.687 |
| JCHAIN | 20.694 |
| CD27 | 2.331 |
| NINJ1 | 16.559 |
| ICAM3 | 0.475 |
| IL2RA | 1.756 |
| CD6 | 1.016 |
| CD3E | 3.985 |
| ICOS | 0.973 |
| TNFRSF8 | 0.432 |
| CXCR3 | 1.105 |
| MMP25 | 0.816 |
| SEC61G | 31.008 |
| LTB | 3.838 |
| CCR7 | 1.566 |
| TIGIT | 0.867 |
| SAP30L | 3.935 |
| LAIR2 | 0.314 |
| CORO1A | 8.031 |
| CPNE5 | 0.911 |
| CD28 | 0.389 |
| CD19 | 0.185 |
| ITGB7 | 0.766 |
| KLRB1 | 0.652 |
| CTTN | 43.56 |
| IL10RA | 2.22 |
| YKT6 | 45.22 |
| ADAMTS1 | 4.321 |
| SMS | 36.038 |
| SBF1 | 14.021 |
| CTSG | 0.596 |
| PKD1 | 3.135 |
| MASP1 | 0.195 |
| TMCO1 | 14.788 |
| TPSAB1 | 3.973 |

**Supplementary Table 1.** Cutoff values for prognostic-related gene

| Dataset | Sample Properties and Number | Data set properties of the model |
| --- | --- | --- |
| TCGA | Tumor samples N=494 | Training set |
| GSE65858 | Tumor samples N=270 | Validation set |
| GSE41613 | Tumor samples N=97 | Validation set |

**Supplementary Table 2.** Sample size statistic

| Age | <60 | 216 |
| --- | --- | --- |
|  | >=60 | 278 |
| Gender | FEMALE | 132 |
|  | FEMALE | 362 |
| Stage | Stage I | 19 |
|  | Stage II | 93 |
|  | Stage III | 102 |
|  | Stage IV | 266 |
| Grade | G1 | 61 |
|  | G2 | 294 |
|  | G3 | 118 |
|  | G4 | 2 |
| Alcohol history | Yes | 327 |
|  | NO | 156 |

**Supplementary Table 3.** Statistical table of clinical information of HNSC cohort samples

| Gene | Forward primer (5′‐3′) | Reverse primer (5′‐3′) |
| --- | --- | --- |
| AREG | GTGGTGCTGTCGCTCTTGATAC | AGGACGGTTCACTACTAGAAGGC |
| CCR7 | GGAGTGAAGGGGCCAAG | GGAGGCCAGAAGGTTCATT |
| CD6 | AGGACGGTTCACTACTAGAAGGC | CTTGATGGATGGAGACCGA |
| CTLA4 | CCCACCGCCATACTACC | CAACCCCGAACTAACTGO |
| TMCO1 | ATTCCATATTTGATGGTAGAGTGG | AACAGTCTGTGGTGTCATCTCCC |
| CTTN | CCGCAGGATCAGGAAACTCA | CCTCAGGAGCAAAAACACGC |

**Supplementary Table 4.** The primer sequences for six genes
